# Rediscovery of *Pogostemon dielsianus* (Lamiaceae, Lamioideae), a rare endemic species from southwestern China, after one century

**DOI:** 10.3897/phytokeys.171.60389

**Published:** 2021-01-12

**Authors:** Guo-Xiong Hu, Ting Su, Ming-Tai An, Xiao-Yu Wang

**Affiliations:** 1 College of Life Sciences, Guizhou University, Guiyang 550025, Guizhou, China Guizhou University Guiyang China; 2 The Key Laboratory of Plant Resources Conservation and Germplasm Innovation in Mountainous Region Ministry of Education, Guizhou University, Guiyang 550025, China Guizhou University Guiyang China; 3 College of Forestry, Guizhou University, Guiyang 550025, Guizhou, China Guizhou University Guiyang China

**Keywords:** Critically endangered, Nujiang Canyon, *Pogostemon elsholtzioides*, *Pogostemon griffithii*, subg. *Pogostemon*, Yunnan

## Abstract

*Pogostemon dielsianus* (Lamiaceae) was described in 1913 based on a single gathering from northwestern Yunnan of China collected in 1905, and thereafter no further collections were observed until 2019. We rediscovered the rare endemic species in Lushui County, Yunnan. Molecular phylogenetic analyses based on four cpDNA markers (*rbcL*, *rps16*, *psbA-trnH*, and *trnL-trnF*) and the nuclear ribosomal internal transcribed spacer (ITS) region confirmed its infrageneric placement within subg. Pogostemon. Based on observations of the rediscovered population of *P. dielsianus*, we updated its morphological description, provided an illustration, and discussed its distribution. Under IUCN criteria, the species was categorized as “Critically Endangered (CR)”.

## Introduction

*Pogostemon* Desf. is the largest genus of tribe Pogostemoneae of subfamily Lamioideae in Lamiaceae (Bendiksby et al. 2011; Li et al. 2016). After combining with *Dysophylla* Blume based on the molecular phylogenetic analyses (Bendiksby et al. 2011; Yao et al. 2015, 2016), *Pogostemon*, as currently circumscribed in a broad sense, contains approximately 80 species. On the basis of molecular and morphological evidence, *Pogostemon* was divided into two subgenera: subg. Pogostemon and subg. Dysophyllus (Bl.) Bhatti & Ingr. ex G. Yao, Y.F. Deng & X.J. Ge (Yao et al. 2016). The genus can be easily distinguished from other Lamiaceae genera by possessing moniliform hairs at the middle of the staminal filaments. *Pogostemon* is distributed mainly in tropical and subtropical regions of Asia with a few species in tropical Africa, Northern Australia, Japan and the Korea Peninsula (Bhatti and Ingrouille 1997; Harley et al. 2004; Yao et al. 2015).

In China, 27 species and two varieties were recorded, of which 10 species and one variety are endemic (Yao et al. 2015; Yao and Ge 2018). *Pogostemon dielsianus* was described in 1913 based on a gathering (*G. Forrest 875*) with two specimens deposited at E and K respectively from Fugong County, northwest Yunnan, China and was not collected again since over the following 100 years. When conducting a taxonomic revision of Chinese *Pogostemon*, Yao et al. (2015) noted that only the type specimens of *P. dielsianus* were examined, and the species was unable to be included in the subsequent molecular phylogenetic analysis (Yao et al. 2016).

During a scientific field trip in Nujiang Canyon, northwestern Yunnan of China in November 2019, a population of *Pogostemon* was discovered in thickets near a tributary of Nujiang River (also known as Salween River). After scrutiny of the data available (Wu and Huang 1977; Li and Hedge 1994; Bhatti and Ingrouille 1997; Yao et al. 2015), we rediscovered *Pogostemon dielsianus* after 106 years. This finding allowed us to update its morphological description, discuss its geographic distribution, assess its conservation status, and infer its phylogenetic position within *Pogostemon*.

## Materials and methods

### Taxon sampling, DNA extraction, amplification and sequencing

Following the latest phylogenetic study (Yao et al. 2016), a total of 28 species (including *Pogostemon dielsianus*) were sampled from both subgenera of *Pogostemon* to explore the phylogenetic position of *P. dielsianus* (Table 1). In addition, three species of its sister genus *Anisomeles* R. Brown were selected as outgroups based on previous studies (Li et al. 2016; Yao et al. 2016). Except for the newly generated sequences of *P. dielsianus*, all other data were downloaded from GenBank.

**Table 1.** Voucher information and GenBank accession numbers for taxa used in this study. *indicates the new sequences, and “–” indicates missing data.

Taxa	Voucher	GenBank accession numbers				
		nrITS	*rbcL*	*rps16*	*trnH-psbA*	*trnL-F*
*Anisomeles heyneana* Benth.		–	–	HQ911589	–	HQ911659
*A. indica* (L.) Kuntze	G. Yao 369 (IBSC)	KR608726	KR608471	KR608595	KR608530	KR608658
*A. malabarica* (L.) R. Br. ex Sims	Fagerlind & Klackenberg 343 (S)	MH456886	–	FJ854013	–	FJ854260
*Pogostemon benghalensis* (Burm. f.) Kuntze	R. G. Troth 677 (US)	–	–	HQ911592	KR608568	HQ911663
*P. amaranthoides* Benth.	J. Chen 668 (KUN)	KR608745	KR608490	KR608614	KR608549	KR608677
*P. aquaticus* (C.H. Wright) Press	Bidgood et al. 3387 (K)	KR608767	KR608527	KR608655	KR608592	KR608717
*P. auricularius* (L.) Hassk.	G. Yao 362 (IBSC)	KR608761	KR608513	KR608638	KR608575	KR608700
*P. barbatus* Bhatti & Ingr.	G. Yao 274 (IBSC)	KR608762	KR608514	KR608639	KR608576	KR608701
*P. brachystachyus* Benth.	G. Yao 358 (IBSC)	KR608775	KR608517	KR608642	KR608579	KR608704
*P. cablin* (Blanco) Benth.	G. Yao 291 (IBSC)	KR608757	KR608503	KR608627	KR608562	KR608690
*P. chinensis* C.Y. Wu & Y.C. Huang	G. Yao 445 (IBSC)	KR608742	KR608512	KR608637	KR608573	KR608699
*P. dielsianus* Dunn	Hu et al 636 (GACP)	MW194872*	MW194874*	MW194875*	MW194873*	MW194876*
*P. elsholtzioides* Benth.	Syn. s.n. (US sheet no. 262106)	–	–	KR608633	KR608569	KR608720
*P. formosanus* Oliver	R. Q. Gao & S. H. Lai 710 (PE)	KR608779	KR608500	KR608624	KR608559	KR608687
*P. fraternus* Miq.	Syn. 7655 (KUN)	KR608781	–	KR608648	KR608585	KR608710
*P. glaber* Benth.	G. Yao 364 (IBSC)	KR608740	KR608496	KR608620	KR608555	KR608683
*P. heyneanus* Benth.	G. Yao 297 (IBSC)	KR608751	KR608492	KR608616	KR608551	KR608679
*P. hispidocalyx* C.Y. Wu & Y.C. Huang	Expedition to QTP 9446 (KUN)	KR608780	–	KR608644	KR608581	KR608706
*P. linearis* (Benth.) Kuntze	G. Yao 348 (IBSC)	KR608764	KR608521	KR608649	KR608586	KR608711
*P. litigiosus* Doan ex Suddee & A. J. Paton	V. D. Nong 31712077 (IBSC)	KR608776	KR608519	KR608645	KR608582	KR608707
*P. macgregorii* W. W. Sm.	K.Iwatsuki et al. 9659 (A)	KR608778	–	–	–	–
*P. paniculatus* (Willd.) Benth.	Middleton et al. 1532 (K)	–	–	–	KR608574	KR608721
*P. paniculatus* (Willd.) Benth.	J. Klackenberg & R. Lundin 565 (S)	–	–	FJ854071	–	
*P. parviflorus* Benth.	G. Yao 365 (IBSC)	KR608749	KR608501	KR608625	KR608560	KR608688
*P. petelotii* Doan ex G. Yao, Y.F. Deng & X.J. Ge	T. Sorensen et al. 6313 (KUN)	KR608772	KR608529	KR608657	KR608594	KR608719
*P. plectranthoides* Desf.	G. Yao 449 (IBSC)	KR608758	KR608510	KR608635	KR608571	KR608697
*P. quadrifolius* (Benth.) F. Muell.	F. G. Dickason 8194(A)	KR608773	KR608518	KR608643	KR608580	KR608705
*P. rogersii* N E. Br.	Phillips 3855 (K)	KR608782	–	KR608647	KR608584	KR608709
*P. sampsonii* (Hance) Press	G. Yao 273 (IBSC)	KR608769	KR608524	KR608652	KR608589	KR608714
*P. septentrionalis* C.Y. Wu & Y.C. Huang	G. Yao 264 (IBSC)	KR608747	KR608497	KR608621	KR608556	KR608684
*P. stellatus* (Lour.) Kuntze	B. Z. Xiao 4826 (K)	KR608768	KR608523	KR608651	KR608588	KR608713
*P. xanthiifolius* C.Y. Wu & Y.C. Huang	H. T. Tsai 59-10586 (KUN)	KR608746	KR608493	KR608617	KR608552	KR608680
*P. yatabeanus* (Makino) Press	G. Yao 285 (IBSC)	KR608766	KR608526	KR608654	KR608591	KR608716

Total genomic DNA of *Pogostemon dielsianus* was extracted from silica gel-dried leaf material following the modified CTAB method of Doyle and Doyle (1987). The nuclear ribosomal internal transcribed spacer (ITS) region was amplified using primers ITS5 and ITS4 (White et al. 1990). Four chloroplast DNA markers were employed to make phylogenetic analyses and the *rbcL* was amplified with primers of Z1F and 51R (Soltis et al. 1992), the *rps16* with rps-LamF and rps-LamR (Bendiksby et al. 2011), the *psbA-trnH* with psbAF and trnHR (Sang et al. 1997), and the *trnL-trnF* with trn-c and trn-f (Taberlet et al. 1991). All makers were amplified and sequenced with the same conditions following Hu et al. (2020).

### Sequence alignment and phylogenetic analyses

Sequences were checked and assembled employing Sequencher v.4.1.4 (Gene Codes, Ann Arbor, Michigan, USA) and then aligned Mafft-win v7.221 (Katoh and Standley 2013) by default. The final alignments were manually adjusted in PhyDE v.0.9971 (Müller et al. 2010). Nuclear dataset (ITS) and plastid matrix (consisting of *rbcL*, *rps16*, *psbA-trnH*, and *trnL-trnF*) were analyzed separately using maximum likelihood (ML) and Bayesian inference (BI). ML analyses were performed using RAxML-HPC2 on XSEDE v.8.2.12 (Stamatakis 2014) under the GTRCAT model on the CIPRES science gateway portal (http://www.phylo.org/) (Miller et al. 2010). Except for setting the bootstrap iterations (-# | -N) to 1000, other parameters followed default. BI analysis was performed in MrBayes v3.2.6 (Ronquist et al. 2012) as implemented in PhyloSuite (Zhang et al. 2020) with the ModelFinder used to select the best model (Kalyaanamoorthy et al. 2017). Under the Akaike information criterion (AIC), the GTR+F+G4 model was selected for nrDNA dataset and the GTR+F+I+G4 for cpDNA matrix. In each analysis, four Markov chain Monte Carlo (MCMC) chains were run simultaneously for 20 million generations, starting with one random tree and sampling one tree every 1000^th^ generation. Convergence of runs was reached when the average standard deviation of split frequencies (ASDSF) fell below 0.01. After discarding the first 25% of the resulting trees as burn-in, the remaining trees were used to assess posterior probabilities (PP) in a majority-rule consensus tree.

## Results and discussion

### Phylogenetic position of *Pogostemon dielsianus*

**Figure 1.**
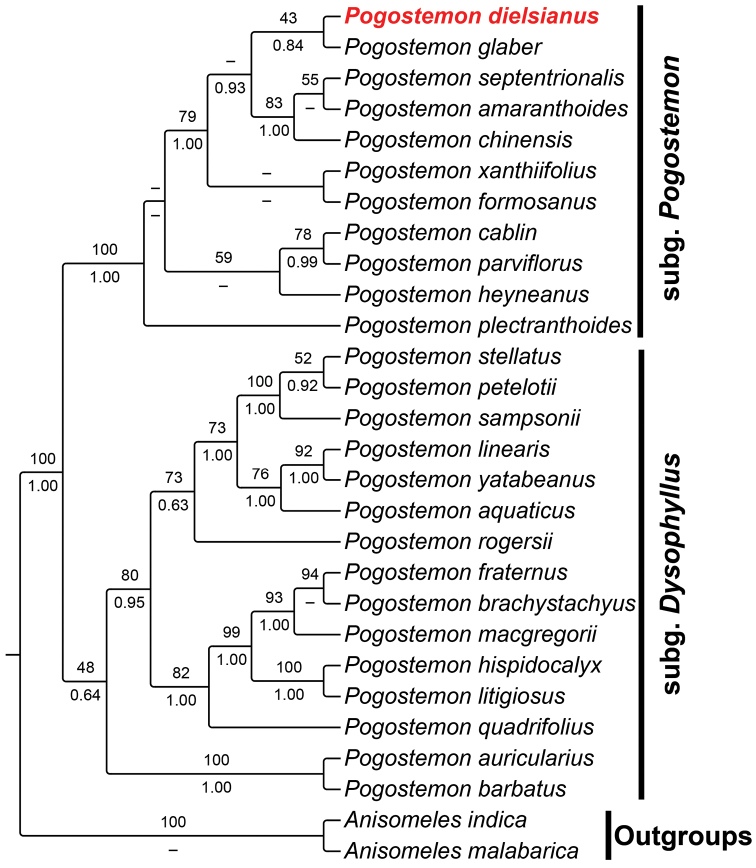
Cladogram of *Pogostemon* based on ML analysis of internal transcribed spacers (ITS) matrix. *Pogostemon dielsianus* is highlighted in red. Bootstrap values of ML are given above the branches with posterior probabilities (PP) of BI below. Bootstrap values <40% and PP< 0.6 are indicated by a dash.

**Figure 2.**
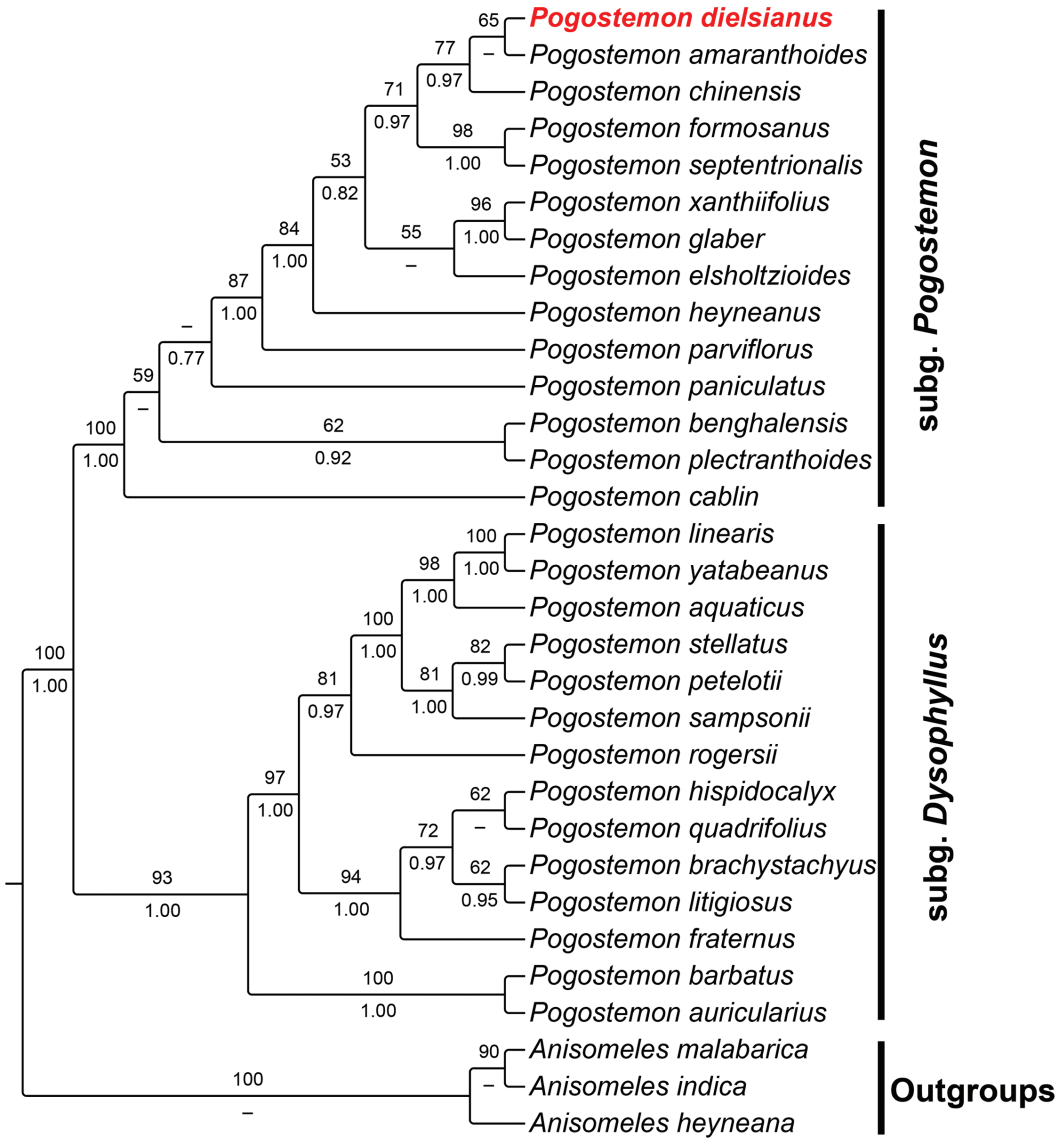
Cladogram of *Pogostemon* based on ML analysis of the combined cpDNA (*rbcL*, *rps16*, *psbA-trnH*, and *trnL-trnF*) dataset. *Pogostemon dielsianus* is highlighted in red. Bootstrap values of ML are given above the branches with posterior probabilities (PP) of BI below. Bootstrap values <40% and PP< 0.6 are indicated by a dash.

Both nrDNA and cpDNA analyses supported the monophylies of *Pogostemon* and its two subgenera (subg. Pogostemon and subg. Dysophyllus). Although *Pogostemon dielsianus* fell into the subg. Pogostemon in both trees, its phylogenetic position was not entirely consistent (Figs 1, 2). In nrDNA topology, *P. dielsianus* seemed to be sister to *P. glaber* Benth., then together sister to the clade consisting of *P. chinensis* C.Y. Wu & Y.C. Huang + *P. septentrionalis* C.Y. Wu & Y.C. Huang + *P. amaranthoides* Benth. (Fig. 1). However, in cpDNA tree, *P. dielsianus*, instead of grouping with *P. glaber*, was sister to *P. amaranthoides*, then together sister to *P. chinensis* (Fig. 2).

Morphologically, *Pogostemon dielsianus* is similar to *P. elsholtzioides* Benth. and *P. griffithii* Prain in having lanceolate leaves (Yao et al. 2015). For the two similar species, only four cpDNA sequences of *P. elsholtzioides* are available in GenBank. In the cpDNA topology, instead of grouping with the morphologically similar species (*P. dielsianus*), *P. elsholtzioides* was sister to the clade consisting of *P. glaber* and *P. xanthiiphyllus* C.Y. Wu & Y.C. Huang. However, due to the unavailability of nrDNA sequences of *P. elsholtzioides*, the conclusion that *P. dielsianus* is not closely related to *P. elsholtzioides* solely on the basis of cpDNA result, cannot be drawn at present. In fact, discordances stemming from nuclear and plastid genomes are common in Lamiaceae, which may be attributed to ancient hybridization with chloroplast capture (Drew and Sytsma 2013; Xiang et al. 2013; Drew et al. 2014; Deng et al. 2015; Hu et al. 2018). Therefore, further studies, especially including nrDNA sequences of *P. elsholtzioides* and *P. griffithii*, are needed to clarify the true phylogenetic position of *P. dielsianus* within subg. Pogostemon.

### Taxonomic treatment

#### Pogostemon dielsianus

Taxon classificationPlantaeLamialesLamiaceae

Dunn in Notes Bot. Gard. Edinburgh 8: 159. 1913.

6B62590E-4466-5397-BE7E-0044E1276162

Figs 3 , 4

##### Lectotype

(designated by Bhatti and Ingrouille in Bull. Nat. Hist. Mus. Lond. (Bot.) 27: 99. 1997). China. Yunnan: Fugong, Valley of the Salween, between Shih-chi-ti and Shia-ku-ti, Salween-Irrawaddy Divide, 26°20'N, 1524–1829 m, November 1905, G. Forrest 875 (E [barcode E00087126, image!]; isolectotype: K [barcode K000249619, image!]).

**Figure 3.**
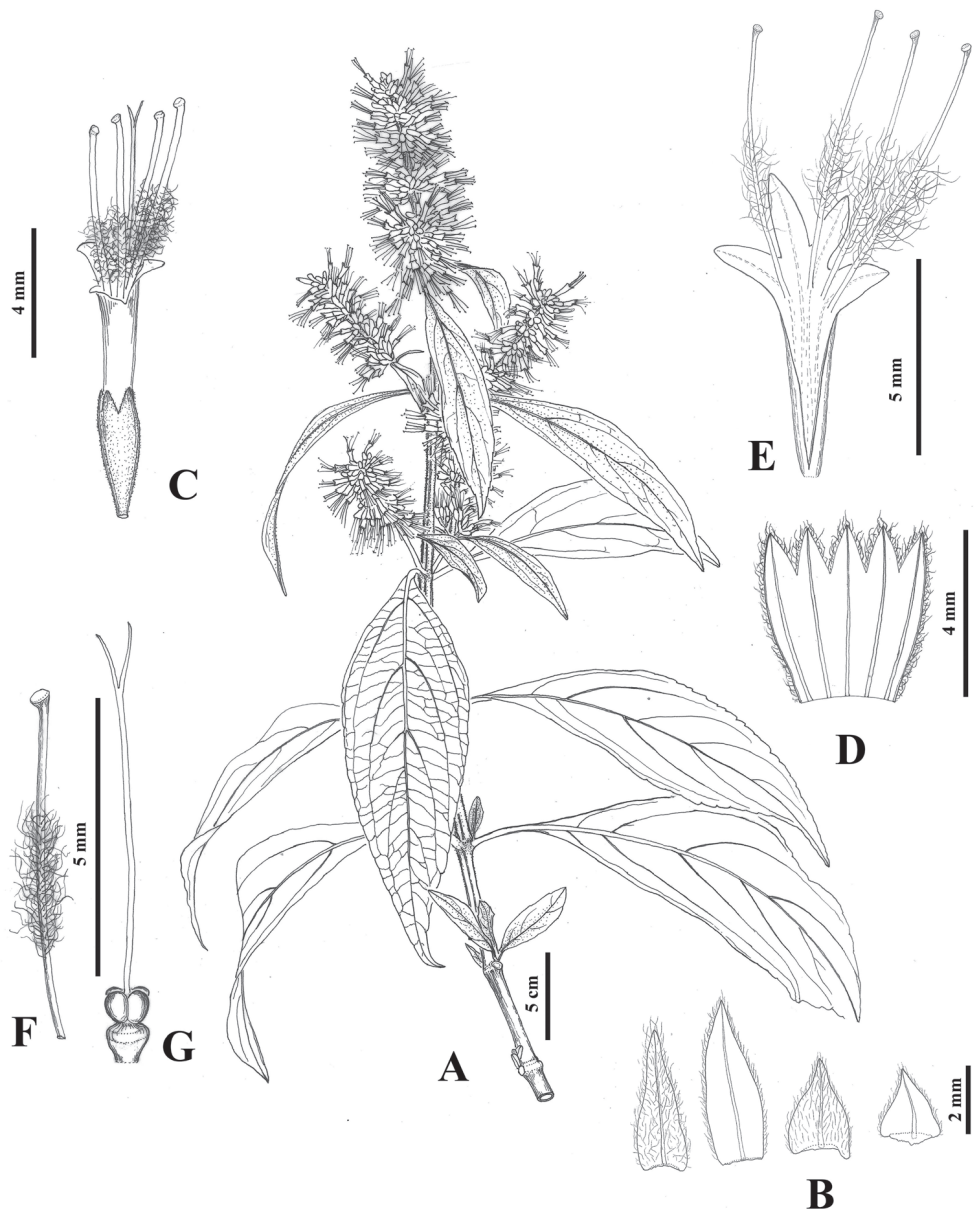
*Pogostemon dielsianus***A** floral branches **B** bract and bracteole **C** flower **D** dissected calyx **E** dissected corolla showing stamens **F** stamen **G** pistil. Drawn by Xiao-Yu Wang based on *Hu et al. 636* (GACP).

##### Revised description.

Perennial shrubs, up to 3 m tall. Stems solid, gray, ground diameter to 3.5 cm, branches terete or angular, slightly dilated at nodes, the initial branches green, densely strigose-pubescent, 2–3-year-old branches yellow-brown, subglabrous. Leaves opposite; petiole 0.5–2.5 cm long; blade linear-lanceolate to lanceolate, 8–14 × 2–4 cm, papery, both sides densely strigose-puberulent when young, the mature gradually subglabrous, base cuneate, margin serrate, apex acuminate, lateral veins 3–6 pairs. Spikes 3.5–7 cm long, 8–12 mm wide, terminal and axillary, subcontinuous, basally somewhat lax, with more than two lateral branches, densely appressed pubescent except for corolla, pedunculate, 0.5–2 cm long; cymes sessile, 8–14-flowered, flowers sessile. Bracts 4–6.5 mm, bracteoles 1.8–2.3 mm. Calyx tubular, 3.5–4.5 mm long, 5-veined; teeth 5, triangular, 1/5–1/4 as long as the calyx tube. Corolla rose, 2-liped, 7–9 mm long, glabrous outside; tube cylindric, dilated at throat, ca. 2× as long as calyx; upper lip 3-lobed, lobes triangular, subequal, 1.1–1.3 × 0.9–1.1 mm; lower lip entire, ca. 0.9 × 0.7 mm. Stamens 4, exserted from corolla; filaments 5.5–7 mm long, exserted portion ca. 3.5 mm. Style 6.3–8.5 mm long; stigma bifid, lobes subequal, 1.1–1.3 mm. Disc ca. 0.7 mm long. Nutlets 4, ca. 1.5 × 0.8 mm, lanceolate.

**Figure 4.**
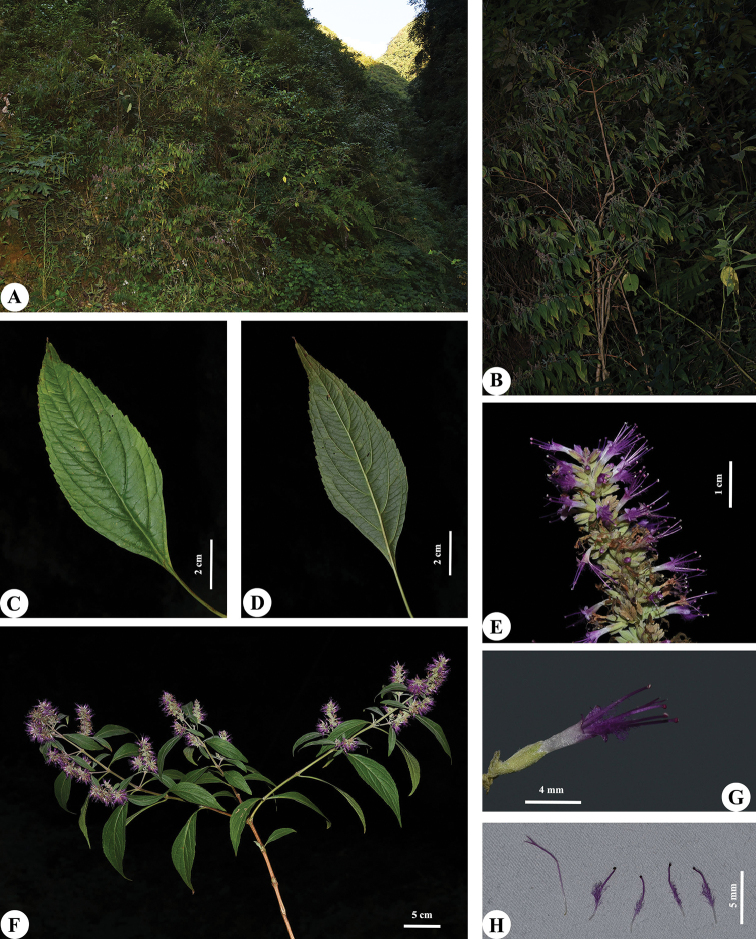
Habitat and morphology of *Pogostemon dielsianus***A** habitat **B** habit **C** leaf, adaxial view **D** leaf, abaxial view **E** inflorescence **F** floral branches **G** flower **H** Stamens and style. Photographed by G.X. Hu.

##### Distribution and habitat.

**Figure 5.**
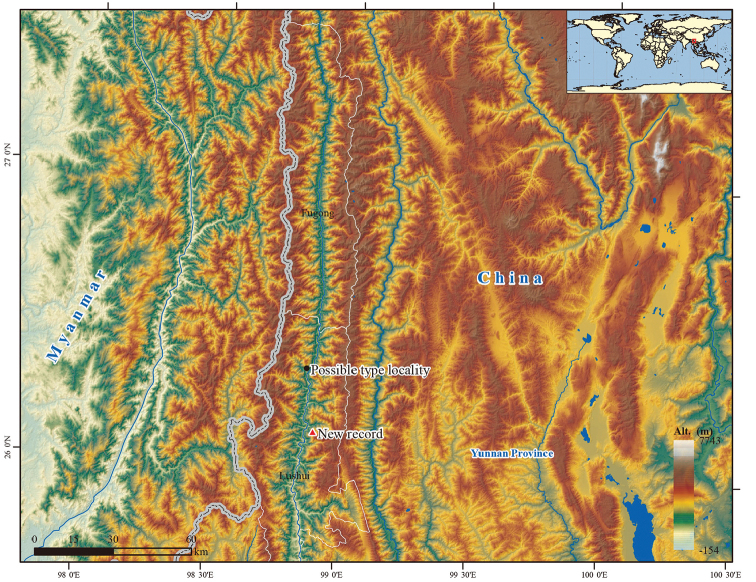
Current distribution of *Pogostemon dielsianus*.

The type locality of *Pogostemon dielsianus* was recorded in Fugong County, northwestern Yunnan of China, which is the only historical known site until our new discovery. As coordinate information of the collection is incomplete due to the lack of longitude data, the precise situation of type specimen is unclear. Based on the latitude provided in the original record, the type specimen is more likely to be collected in the north of Lushui County, a neighboring county of Fugong (Fig. 5). Although the recently collected population was also discovered in north Lushui County, distribution of the two populations does not overlap because they are located on different sides of Nujiang River (Fig. 5). In accordance with type specimen record, *P. dielsianus* grows amongst thickets on dry rocky hillsides with elevations ranging from 1524–1829 m. The finding that the newly recorded population grows on the riverside indicates that *P. dielsianus* is more likely to occur in humid areas of dry hillsides. Actually, a similar habitat can also be found elsewhere in Nujiang Canyon. Potential populations of this species, therefore, may be discovered through further field investigation in this region.

##### Phenology.

Flowering and fruiting from November to December.

##### Conservation status and preliminary IUCN assessment.

*Pogostemon dielsianus* is historically known from only two specimens collected from the type locality (Fugong, Yunnan, China) in 1905, and it has not been recollected for the past 114 years until our expedition to Nujiang Canyon in 2019. In the newly recorded locality (Lushui, Yunnan, China), only about 10 mature individuals have been discovered. Due to the lack of exact geographical information of the type locality, it is difficult to confirm the number of individuals there. Based on current investigations and historical records, we inferred that mature individuals of this species may be fewer than 250, and no subpopulation contains more than 50 mature individuals. Therefore, under the IUCN criteria C2a(i) (IUCN 2012), we propose that *P. dielsianus* should be classified as “Critically Endangered (CR)”.

##### Additional specimens examined.

China. Yunnan: Lushui County, Daxingdi Town, Tuanjie Village, Luchuluo, amongst a thicket near the Luchuluo River, elevation 1786 m, 26°7.14'N, 98°53.78'E, 24 November 2019, *Hu et al. 636* (GACP!, IBSC!, KUN!).

##### Notes.

*Pogostemon dielsianus* is morphologically similar to *P. elsholtzioides* and *P. griffithii* in having lanceolate leaves. However, *P. dielsianus* can be easily distinguished from *P. elsholtzioides* and *P. griffithii* by its longer and tubular calyx, smaller ratio of the length of calyx teeth and calyx tube and longer corolla, filament and style (Table 2). In addition, the geographical distribution of these three species is also different in that *P. dielsianus* is endemic to NW Yunnan, China, *P. elsholtzioides* is widely distributed in the Himalayan regions (Bhutan, India, and SE Xizang, China), and *P. griffithii* is endemic to Myanmar (Bhatti and Ingrouille 1997; Yao et al. 2015; Yao and Ge 2018).

**Table 2.** Morphological comparison between *Pogostemon dielsianus* and its morphologically similar species.

Character	*Pogostemon dielsianus*	*Pogostemon elsholtzioides*	*Pogostemon griffithii*
Calyx	tubular, 3.5–4.5 mm long	campanulate, 3–3.5 mm long	campanulate, ca. 3.5 mm long
Ratio of the length of calyx teeth and calyx tube	1/5–1/4	1/3–1/2	1/2–1
Corolla length	7–9 mm	ca. 4.5 mm	ca. 5 mm
Filament length	6.2–7 mm	4.5–5 mm	4.7–5.2 mm
Style length	6.3–8.8 mm	ca. 5.5 mm	ca. 5.5 mm
Nutlet	lanceolate	lanceolate	oblong
Distribution	China (NW Yunnan)	Bhutan, India, China (SE Xizang)	Myanmar

In the protologue, Dunn (1913) did not designate a type for the name *Pogostemon dielsianus* Dunn. Bhatti and Ingrouille (1997) indicated the specimen deposited in E and K as holotype and isotype, respectively. In fact, they effectively chose the lectotype for the name and the term“holotype” and “isotype” can be corrected as “lectotype” and “isolectotype” according to Article 9.10 of the International Code of Nomenclature for algae, fungi, and plants (Shenzhen Code) (Turland et al. 2018).

## Supplementary Material

XML Treatment for Pogostemon dielsianus
